# A Platelet-Rich Plasma-Derived Biologic Clears *Staphylococcus aureus* Biofilms While Mitigating Cartilage Degeneration and Joint Inflammation in a Clinically Relevant Large Animal Infectious Arthritis Model

**DOI:** 10.3389/fcimb.2022.895022

**Published:** 2022-05-30

**Authors:** Jessica M. Gilbertie, Thomas P. Schaer, Julie B. Engiles, Gabriela S. Seiler, Bennett L. Deddens, Alicia G. Schubert, Megan E. Jacob, Darko Stefanovski, Gordon Ruthel, Noreen J. Hickok, Devorah M. Stowe, Alexa Frink, Lauren V. Schnabel

**Affiliations:** ^1^ Department of Clinical Sciences, College of Veterinary Medicine, North Carolina State University, Raleigh, NC, United States; ^2^ Comparative Medicine Institute, North Carolina State University, Raleigh, NC, United States; ^3^ Department of Clinical Studies New Bolton Center, School of Veterinary Medicine, University of Pennsylvania, Kennett Square, PA, United States; ^4^ Department of Pathobiology New Bolton Center, School of Veterinary Medicine, University of Pennsylvania, Kennett Square, PA, United States; ^5^ Department of Molecular Biomedical Sciences, College of Veterinary Medicine, North Carolina State University, Raleigh, NC, United States; ^6^ Department of Population Health and Pathobiology, College of Veterinary Medicine, North Carolina State University, Raleigh, NC, United States; ^7^ Department of Orthopedic Surgery, Sidney Kimmel Medical College of Thomas Jefferson University, Philadelphia, PA, United States

**Keywords:** infectious arthritis, *S. aureus*, biofilm, antimicrobial, platelets, cartilage, synovium, synovial fluid

## Abstract

The leading cause of treatment failure in *Staphylococcus aureus* infections is the development of biofilms. Biofilms are highly tolerant to conventional antibiotics which were developed against planktonic cells. Consequently, there is a lack of antibiofilm agents in the antibiotic development pipeline. To address this problem, we developed a platelet-rich plasma (PRP)-derived biologic, termed BIO-PLY (for the BIOactive fraction of Platelet-rich plasma LYsate) which has potent *in vitro* bactericidal activity against *S. aureus* synovial fluid free-floating biofilm aggregates. Additional *in vitro* studies using equine synoviocytes and chondrocytes showed that BIO-PLY protected these cells of the joint from inflammation. The goal of this study was to test BIO-PLY for *in vivo* efficacy using an equine model of infectious arthritis. We found that horses experimentally infected with *S. aureus* and subsequently treated with BIO-PLY combined with the antibiotic amikacin (AMK) had decreased bacterial concentrations within both synovial fluid and synovial tissue and exhibited lower systemic and local inflammatory scores compared to horses treated with AMK alone. Most importantly, AMK+BIO-PLY treatment reduced the loss of infection-associated cartilage proteoglycan content in articular cartilage and decreased synovial tissue fibrosis and inflammation. Our results demonstrate the *in vivo* efficacy of AMK+BIO-PLY and represents a new approach to restore and potentiate antimicrobial activity against synovial fluid biofilms.

## Introduction

Infectious, or septic, arthritis is a life-threating orthopedic emergency that requires rapid diagnosis and prompt treatment (Shirtliff and Mader, 2002; Tong et al., 2015). These treatments rely on systemic and local antimicrobial therapy, combined with surgical debridement and irrigation (Davis, 2005; Sharff et al., 2013). Nevertheless, even if the infection is cleared, chronic inflammation can persist, resulting in substantial damage to the joint and culminating in degenerative joint disease or osteoarthritis (Shirtliff and Mader, 2002; Tong et al., 2015). Thus, while many treatments have focused on agents that inhibit vital functions of bacteria like quorum sensing (Zimmerli and Moser, 2012; Brackman and Coenye, 2015), curing the infection alone is inadequate to deal with the total disease pathology of infectious arthritis.

*Staphylococcus aureus* is the most common bacterial isolate in all forms of osteoarticular infections including infectious arthritis and periprosthetic joint infection (PJI) and is associated with treatment failure rates of up to 72% (Byren et al., 2009; Cobo et al., 2011). These high treatment failure rates are strongly associated with the propensity of Staphylococci to form biofilms (Zimmerli and Moser, 2012; McConoughey et al., 2014) which cloak bacteria from the host immune system and offer protection from antimicrobial therapies (Leid et al., 2002; Høiby et al., 2010; Stoodley et al., 2011; Moser et al., 2017; Ciofu et al., 2022). Importantly, while biofilms form on implanted hardware, free-floating bacterial aggregates also readily form in synovial fluid (SF) (Dastgheyb et al., 2015; Gilbertie et al., 2019). The presence of these highly antibiotic-tolerant aggregates is thought to be one of the reasons for the difficulties in treating infectious arthritis.

Bacterial infiltration into a joint leads to the production of inflammatory mediators such as IL-1β, TNF-α, and IL-8 (Shirtliff and Mader, 2002). Thereafter, an influx of immune cells, mainly neutrophils (>80%), into the synovial membrane and SF can result in the production of metalloproteases and other catabolic enzymes (Boff et al., 2018). The result of this inflammatory cascade is increased arthritic changes such as cartilage erosion, osteophyte formation, proteoglycan loss, chondrocyte death and chondrone formation amongst other changes in extracellular matrix composition (McIlwraith and Vachon, 1988).

To address both active bacterial infection and subsequent tissue damage, we considered the use of platelet-rich plasma (PRP). PRP is a widely used therapeutic for musculoskeletal injuries and has recently been explored for its antibacterial properties (Gato-Calvo et al., 2019; Zhang et al., 2019). The activities attributed to PRP, however, appear to be dependent on both donor properties and preparation method leading to variable activity (Giraldo et al., 2013; Xiong et al., 2018). To allow for increased reproducibility, we pooled PRP and isolated the antimicrobial, anti-inflammatory fraction, which was derived from an acellular PRP lysate enriched for <10 kDa cationic peptides (termed BIO-PLY, standing for BIOactive fraction of Platelet-rich plasma LYsate). BIO-PLY was potently antimicrobial against both gram-positive and gram-negative biofilm aggregates and synergized with aminoglycosides to reduce bacterial numbers by 6-7 log in SF (Gilbertie et al., 2020). Furthermore, BIO-PLY mitigated equine synoviocyte and chondrocyte inflammation *in vitro* (Gilbertie et al., 2018).

The purpose of this study was to test *in vivo* efficacy of BIO-PLY using an equine model of *Staphylococcus aureus* infectious arthritis. We chose the horse as a clinically relevant large animal model as A) horses have very similar cartilage biology to humans (Wayne Mcilwraith et al., 2011; McCoy, 2015) and are approved by the FDA as a pre-clinical model for osteoarthritis; B) the equine immune and inflammatory response is similar to that of humans (Horohov, 2015); C) SF can be obtained repeatedly in large quantities from horses using mild sedation without the need for general anesthesia (Maher et al., 2014); and D) horses suffer from naturally occurring infectious arthritis that requires clinical treatment and rehabilitation protocols (Gilbertie et al., 2018). Given these advantages, the horse qualifies for the Dual Purpose with Dual Benefit status with the NIH, benefitting agricultural and biomedical research.

In this study, we combined BIO-PLY with the aminoglycoside amikacin (AMK) as intra-articular injection of aminoglycosides is performed in both the human and veterinary clinical setting for the treatment of joint infections (Whiteside and Roy, 2017). We measured the effect of BIO-PLY on reduction of clinical signs of septic arthritis, bacterial burden, immune cell presence, inflammatory cytokine and other biomarker content, and histological assessments of synovial tissue and cartilage. Our hypothesis was that AMK+BIO-PLY treatment would decrease bacterial burden and improve outcome parameters in an equine *S. aureus* infectious arthritis model compared to horses treated with AMK alone.

## Materials and Methods

### BIO-PLY Preparation

BIO-PLY was prepared from eight healthy horses (4 geldings and 4 mares between the ages of 6 and 19 years). The Institutional Animal Care and Use Committee of North Carolina State University (16-189-O) approved the use of these horses for BIO-PLY preparation. Whole blood was collected *via* jugular venipuncture into syringes containing acid citrate dextrose (ACD). Erythrocytes were removed by sedimentation and the layer above the erythrocytes or leukocyte-rich platelet-rich plasma was centrifuged at 250g for 15 minutes to remove leukocytes. The supernatant or platelet-rich plasma (PRP) was then centrifuged at 1500g for 15 minutes to pellet the platelets. The platelet-poor plasma (PPP) or supernatant above the platelet pellet was saved and the platelet pellet was re-suspended in PPP to generate a 50× PRP. The importance of this plasma component for antimicrobial activity has been previously described (Drago et al., 2014; López et al., 2014; Gilbertie et al., 2020). Leukocyte, erythrocytes, and platelet concentrations were determined as previously described (Gilbertie et al., 2020). The 50× PRP contained greater than 1,000,000 platelet/µL, less than 100 WBC/µL and <10 RBC/µL. PRP lysate (PRP-L) was generated from the 50x PRP by five freeze/thaw cycles. The platelet debris was removed from PRP-L by centrifugation at 20,000g for 20 minutes. Removal of anionic components was performed by incubation with a washed, loose anion exchange resin (UNOsphere Q resin, Bio-Rad Laboratories, Hercules, CA, USA) and subsequent separation of unbound components (cationic and neutral) using a bottle-top filter (0.22µm PES, Nalgene). Fractionation by molecular weight was performed with a 10kDa molecular weight cutoff filter (Amicon^®^ Ultra 15 mL Centrifugal Filters, 10kDa, MilliporeSigma, Burlington, MA). The filtrate containing proteins and peptides <10kDa in size was collected, aliquoted into 5mL aliquots, and stored at -80°C until used in this study. *In vitro* anti-biofilm efficacy was confirmed prior to use in this study (Gilbertie et al., 2020).

### Induction and Treatment of Infection Experimental Design

**Figure 1:**
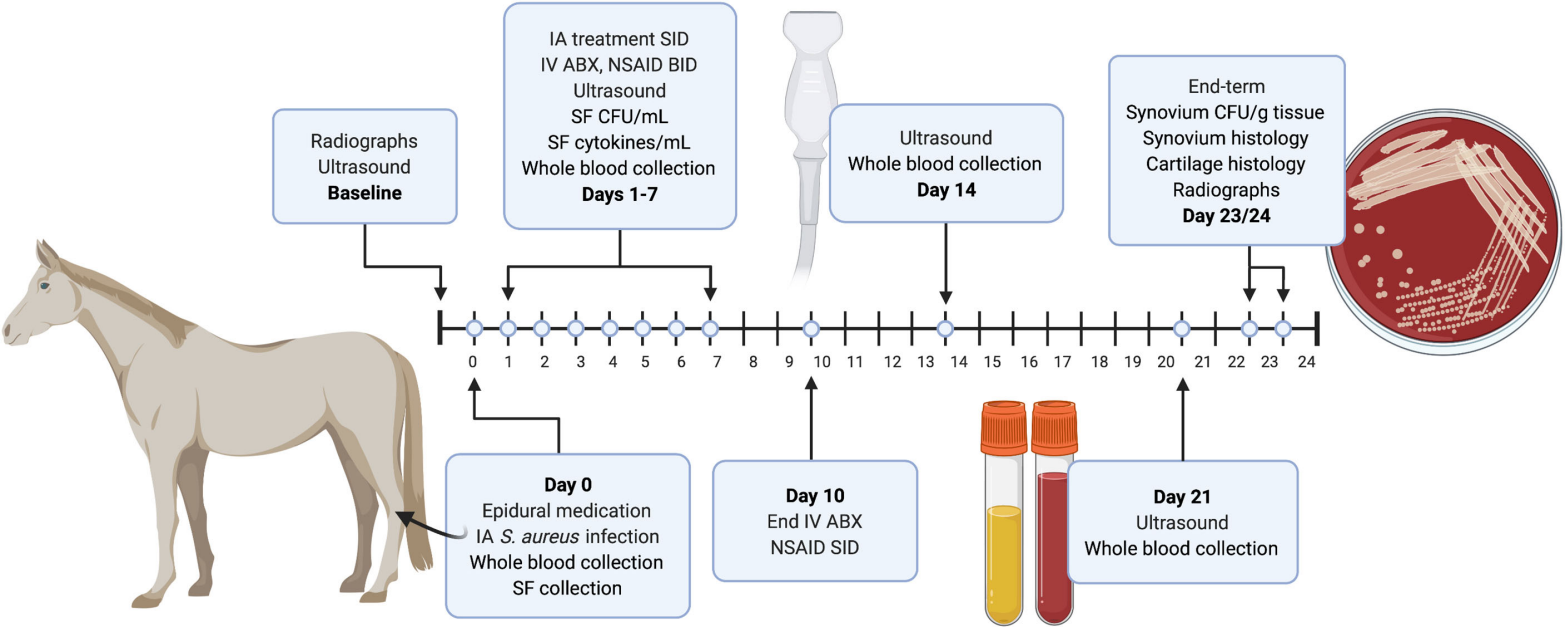
Diagram of the experimental design and timing of major events such as sample collection and ultrasound. Baseline diagnostic imaging and epidural catheter placement was performed 24-48 hours prior to the start of the study. Baseline radiographs and ultrasound images were taken 3-5 days before the start of the study (day 0). On day 0, samples (blood and synovial fluid) were collected, epidural medication was administered, and *S. aureus* was injected intra-articularly (IA) into one randomly chosen tarsocrural joint. Beginning 24 hours post-infection (day 1), horses were treated with 500mg amikacin alone (control AMK; n=6) or BIO-PLY and 500mg of amikacin (AMK+BIO-PLY treatment; n=6) daily for 7 days. Blood and synovial fluid (SF) samples were collected at baseline (day 0) and from days 1-7. Synovial fluid was processed to determine bacterial load (CFU/mL) and cytokine milieu. Whole blood was submitted for in-house complete blood counts and plasma was saved for systemic biomarker analysis. All horses received intravenous (IV) antimicrobials (ABX) and analgesics (NSAID) twice per day (BID) for 7-10 days. From day 7 to 21, NSAIDs were administered once per day (SID). Ultrasound exams were performed at day 14 and 21. At end-term (day 23-24), horses had post-infection radiographs performed, were euthanized, and had samples (synovium and cartilage) collected at autopsy for microbiology and histopathology. Synovium was processed for bacterial load (CFU/g). Synovium and cartilage were harvested and submitted for histopathology.

Skeletally mature horses (n=12; 7 mares, 4 geldings, 1 stallion; ages 2-14 years) with normal physical examinations, bloodwork, and tarsocrural radiographs were randomly allocated into treatment (AMK+BIO-PLY) or control (AMK) groups (IACUC protocol #16-194 of NC State University). A graphical abstract of the study design is presented in **Figure 1**. Horses were quarantined for 14 days and stall acclimated for 5-7 days before entrance into the study. On day 0, horses underwent standing sedation with intravenous detomidine (0.005-0.01mg/kg) and butorphanol (0.005-0.01 mg/kg) to administer epidural analgesia and perform the tarsocrural joint inoculation. A sacrocaudal epidural injection of buprenorphine (0.005 mg/kg) and detomidine (0.01 mg/kg) was performed using a 20-gauge 3.5-inch spinal needle for hindlimb pain control (Fischer et al., 2009). *S. aureus* (ATCC 25923) was prepared by diluting a 0.5 McFarland (1x10^8^ CFU/mL) to yield an inoculum of 1x10^6^ CFU/mL in sterile saline. The final inoculum suspension was then serially diluted, and plate counted to verify the bacterial concentration. Each horse received a 1mL *S. aureus* inoculum (1x10^6^ CFU) *via* intra-articular injection into one randomly assigned tarsocrural joint using a standard dorsomedial approach and a 21-gauge 1.5-inch hypodermic needle. Prior to withdrawal of the needle from the joint, an additional 1mL of sterile saline was injected into the joint to limit *S. aureus* extravasation. Starting 24 hours post-infection, horses were treated with BIO-PLY (5mL) and 500mg amikacin (treatment or AMK+BIO-PLY group; n=6) or 500mg of amikacin and sterile saline (5mL) (control or AMK group; n=6) daily for 7 days. All horses received systemic antimicrobials by intravenous administration of potassium penicillin (22,000 U/kg every 6 hours) in combination with gentamicin (6.6 mg/kg every 24 hours) for 10 days post-infection as well as a tapering course of phenylbutazone (4.4 mg/kg every 12 hours for days 1-3, 2.2 mg/kg every 12 hours for days 4-10 and 2.2 mg/kg every 24 hours for the duration of the study). The minimum inhibitory concentration of *S. aureus* ATCC 25923 for these antimicrobials as measured by antimicrobial susceptibility testing using the Sensititre Complete Automated AST System and the equine (Equine EQUIN1F Vet AST Plate) was measured to be <4 ug/mL for amikacin, ≤ 0.06 ug/mL for penicillin; and ≤1 ug/mL for gentamicin (Gilbertie et al., 2019).

### Clinical Observations

Horses were examined twice daily by a veterinarian for comfort and any clinical signs of systemic infection or sepsis (change in general demeanor, fever, etc.). Horses were evaluated and scored daily for pain with each category scored on a scale of 0-3 (0 = most normal, 3 = most abnormal): lameness, tarsocrural joint swelling, limb edema, pain to palpation of the joint, and heat at the site of infection.

### Sample Collection

Starting on day 0, whole blood, plasma and serum samples were collected. After day 7, blood was collected weekly until the end of the study. Whole blood and serum were analyzed for changes in white blood cell populations and biochemistry values using the Clinical Pathology Laboratory at NC State University College of Veterinary Medicine. SF was aspirated prior to treatment. Portions of the SF samples collected daily were submitted for analysis of total protein, total nucleated cell count and differential cell count. A portion of that sample was processed as a cytospin as well for further cellular analyses. Aliquots of clarified SF were also saved for biomarker/biochemical analysis at the conclusion of the study. All samples were stored at -80°C until analysis.

### Diagnostic Imaging

Horses had pre-infection radiographs performed followed by end-term radiographs at 21 days post-infection. Ultrasonography was performed at day 0 (pre-infection), day 1 (post-infection, pre-treatment), and days 7, 14, and 21 (post-treatment). Tarsocrural joints were imaged with grayscale using the Aplio 500 system (Canon Medical Systems, CA, USA) with a linear 12 MHz broadband transducer. Transverse and longitudinal grayscale images of the dorsomedial and plantarolateral recesses of the tarsocrural joint (**Supplementary Figure 1**) were acquired with the limb weight bearing. All images were anonymized, randomized prior to analyses, assessed independently by two blinded radiologists using established criteria for infectious arthritis (Beccati et al., 2015), and scores were averaged. Degree of distension, synovial thickening, fibrinous loculation, and vascularity as visualized with power Doppler were scored on a scale of 0-3 (0 = most normal, 3 = most abnormal). Character of synovial effusion and presence of hyperechoic foci were scored on a scale of 0-1 (0 = anechoic or absent, 1 = echogenic or present). Radiographs were evaluated using a previously published scoring system for osteoarthritis on a scale of 0-4 (0 = normal, 4 = severe change) evaluated on the basis of: bone proliferation at the joint capsule attachment; subchondral bone lysis; subchondral bone sclerosis; and osteophyte formation (Frisbie et al., 2013).

### Necropsy

At the conclusion of the study, horses were euthanized with an overdose of pentobarbital sodium administered intravenously following sedation according to AVMA Guidelines for the Euthanasia of Animals: 2020 Edition. The infected tarsocrural joint was aseptically prepared and dissection of the tarsocrural joint was performed aseptically from the dorsomedial aspect. The joint was evaluated for gross morphology and photographed as a reference. Synovial membrane samples were collected from four different sites within the joint (dorsomedial, dorsolateral, plantaromedial, and plantarolateral; **Supplementary Figure 1**) for histological and microbiological analysis. Four osteochondral samples were taken from both the lateral and medial trochlea of the talus from the dorsal and plantar aspects for histological analysis.

### Histology

Synovial and osteochondral tissues harvested at necropsy were fixed in neutral-buffered 10% formalin, paraffin embedded, microtome sectioned at 5μm thickness and staining for histologic examination using bright field microscopy and scoring using a modified OARSI system (McIlwraith et al., 2010). Osteochondral tissues were demineralized in an aerated 10% formic acid solution prior to trimming into cassettes. Synovial tissue specimens were stained with Hematoxylin & Eosin (H&E) to assess inflammatory lesions and immunohistochemical (IHC) stains including CD3 for T lymphocytes (DAKO), CD20 for B lymphocytes (Thermo Scientific), CD204 for dendritic cells and macrophages (TransGenic Inc.), and MAC387 (i.e., S100A9, MRP-14, calprotectin) for myeloid leukocytes (i.e., neutrophils and monocytes/macrophages) (DAKO) to further characterize inflammatory cell infiltrates. QuPath (Bankhead et al., 2017) was used for quantitative analyses of positive cell counts identified with each IHC marker from two sections representing the synovial superficial intimal layers including adherent exudate (if applicable) and deeper subintimal regions. MAC387+ cells were further evaluated for morphologic features including cell size and nuclear morphology (i.e., polysegmentation) to determine relative numbers of polymorphonuclear granulocytes (i.e., neutrophils) and monocytes/macrophages within synovial sections. Osteochondral sections were stained with H&E and Safranin O (SafO). Assessment and scoring of histology specimens were performed by a blinded observer. MetaMorph^®^ image analysis software (Molecular Devices Corporation, San Jose, CA, USA) was used for colorimetric quantification to quantify percentage SafO staining within total areas (mm^2^) of non-mineralized articular cartilage within osteochondral sections.

### Microbiology

Historically, it has been difficult to isolate and culture bacteria from the SF of patients with infectious arthritis (Gallo et al., 2008; Taylor et al., 2010). Therefore, we enzymatically digested SF to disperse *S. aureus* biofilm aggregates and quantify bacterial numbers in infected SF (**Supplementary Figure 2**). In brief, 10mL of SF was treated with 200µg/mL proteinase K (QIAGEN, Hilden, Germany) for 1 hour before centrifugation at 300g for 10 min to remove host cells. The supernatant containing SF and dispersed bacteria from SF biofilms was then serially diluted and spot plated in triplicate at each serial dilution for colony counts. At end-term, synovial tissue was removed aseptically at necropsy and weighed in grams. Tissue was then gently homogenized and enzymatically digested for 1 hour in media containing 200µg/mL proteinase K and 1.5mg/mL collagenase type II (ThermoFisher Scientific). Tissue homogenates were then centrifuged at 300g for 15 min to remove host cells and debris and the supernatant containing dispersed bacteria was then serially diluted and spot plated in triplicate at each serial dilution for colony counts. A 1mL aliquot of either the SF or tissue homogenate was also enriched in Thioglycolate broth. The lower limit of detection for this method of bacterial enumeration is 16.7 CFU/mL or 3.4 CFU/g. We reported 0 CFU/mL or CFU/g tissue if all three spots had no visible colonies and the enrichment broth yielded no growth.

### Serum and Synovial Fluid Biomarker Analysis

Venous blood samples were used to determine concentrations of D-Dimer (Shahi et al., 2017) and serum amyloid A (Jacobsen et al., 2006) using the MILLIPLEX MAP Human Cardiovascular Disease (CVD) Magnetic Bead Panel (MilliporeSigma, MA, USA) that the manufacturer predicted to have cross reactivity with equine samples. Concentrations of the predominate inflammatory cytokines found in SF were quantified with the MILLIPLEX MAP Equine Cytokine/Chemokine Magnetic Bead Panel (MilliporeSigma, MA, USA).

### Statistical Analysis

All statistical analyses were performed using Stata 14.1MP, StataCorp, College Station TX, with two-sided tests of hypotheses and a p-value < 0.05 as the criterion for statistical significance. Descriptive analyses include computation of means (with 95% confidence intervals [95%CI]), standard deviations, medians, interquartile ranges (IQR) of continuous variables and tabulation of categorical variables. Tests of normal distribution were performed to determine extent of skewness. Frequency counts and percentages were used for summarizing categorical variables (e.g., sex, signalment and others). As part of the exploratory analysis, principal component analysis was performed to examine intrinsic clusters and obvious outliers within the observations. Discriminant analysis was conducted to establish a set of biomarker concentrations based on variable selection to distinguish between groups. All scores (imaging and histology) were compared between control and treatment groups using Wilcoxon rank-sum tests. Continuous data from ultrasonography, biomechanical testing and cytokine analyses was compared between control and treatment groups using t-tests or non-parametric tests based on normality. Inference statistical analysis was based on a generalized linear mixed model. Sex and other covariates were included as fixed effects in the model where indicated. Systemic and SF cell parameters and biomarkers, and bacterial load were analyzed using multivariate statistical methods. *Post-hoc* pairwise comparisons were used to assess the influence of group.

## Results

### AMK+BIO-PLY Treatment Reduced Bacterial Burden and Decreased Signs of Infection

**Figure 2:**
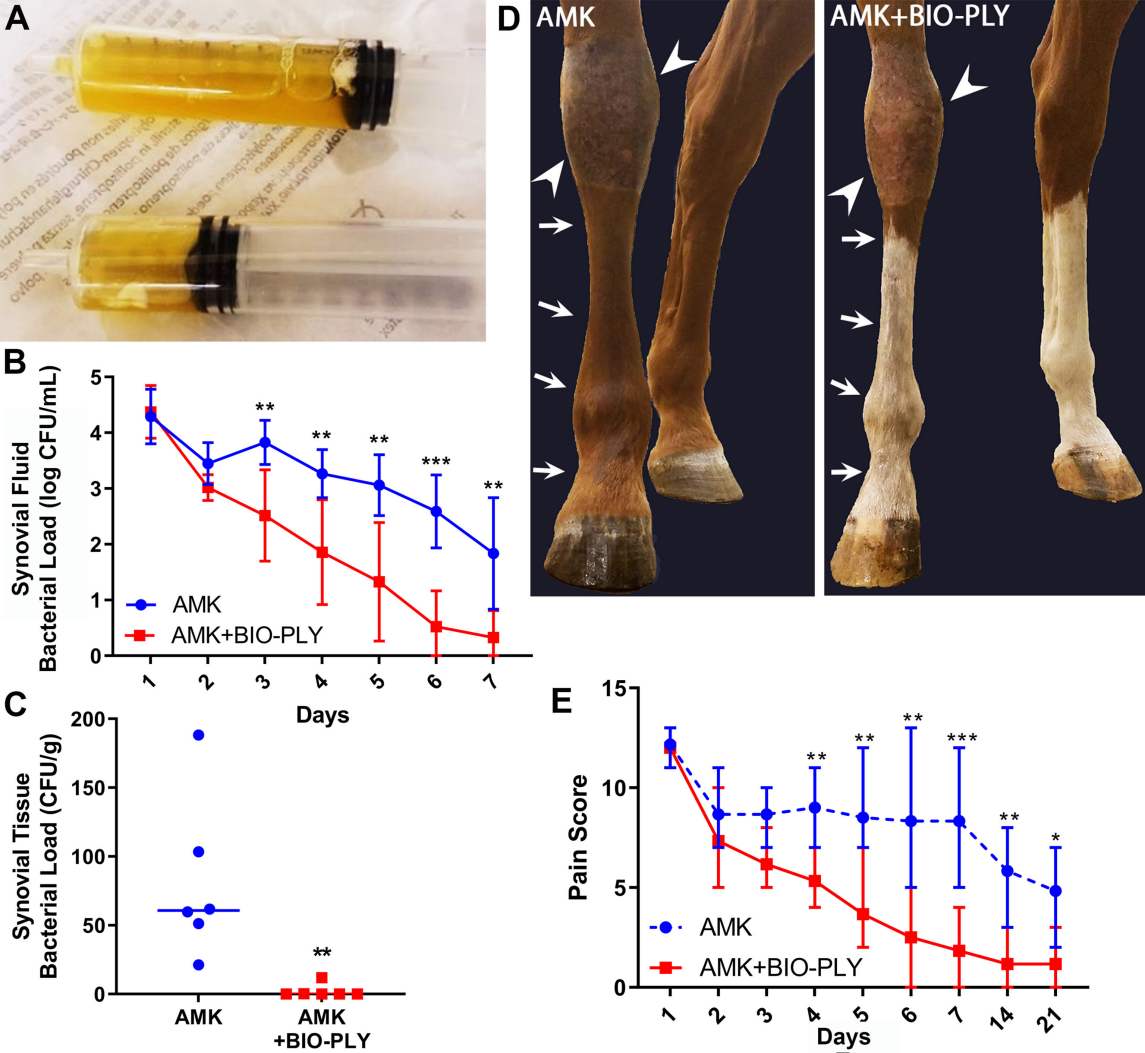
AMK+BIO-PLY treatment decreased bacterial load in the synovial fluid and synovial tissue and reduced clinical signs of infection. **(A)** Synovial fluid biofilm aggregates were observed macroscopically in all horses prior to treatment on day 1. **(B)** Synovial fluid bacterial load was reported as log colony forming units (CFU) per milliliter (mL). **(C)** Bacterial load in the synovial tissue reported as CFU per gram (g). **(D)** Representative photographs of control and treatment limbs evaluated for joint effusion (arrowheads) and limb edema (arrows). Unaffected contralateral limbs are included for each horse as a normal comparison. **(E)** Total pain scores as evaluated for clinical signs of infection for control and treatment limbs where 0 = most normal. Means and standard deviations of each group (control vs treatment; n=6), and significant differences *p<0.05, **p<0.01, ***p<0.001 were determined by the Wilcoxon rank-sum test comparing control and treatment at each day.

At day 1 post-infection, all horses had macroscopically visible SF biofilm aggregates within their experimentally infected joints (**Figure 2A**). *S. aureus* concentrations were measured within their SF (**Figure 2B**) after dispersal of aggregates (**Supplementary Figure 2**). Over the 7 days of treatment, SF from both AMK and AMK+BIO-PLY treated joints showed a time-dependent decrease in SF bacterial counts. By day 3, AMK+BIO-PLY treated joints had significantly fewer bacteria within the SF (2.52 ± 0.82 log CFU/mL) compared to horses treated with AMK alone (3.82 ± 0.40 log CFU/mL) (p<0.005; **Figure 2B**). At end-term, AMK+BIO-PLY treated horses had significantly fewer bacteria within the synovial tissue (2.06 ± 4.86 CFU/g tissue) compared to horses treated with AMK alone (80.95 ± 58.77 CFU/g tissue) (p<0.009; **Figure 2C**). Notably, 5 out of 6 AMK+BIO-PLY treated synovial tissues showed complete bacterial clearance (based on the detection limits of our assay) and the 1 out of 6 that did not show complete bacterial clearance showed a drastically reduced bacterial load.

AMK treated limbs showed persistent swelling above and immediately at the level of the fetlock (**Figure 2D**). This swelling was not apparent at day 7 in the AMK+BIO-PLY treated limbs. The pain score, based on lameness, tarsocrural swelling, limb edema, pain to palpation of the joint, and heat at the site of infection decreased for all horses over time (**Figure 2E**). Notably, by day 4, AMK+BIO-PLY treated horses had significantly lower pain scores compared to AMK treated horses (p<0.008).

### AMK+BIO-PLY Treatment Improved Ultrasonographic Appearance of Tarsocrural Joints

**Figure 3:**
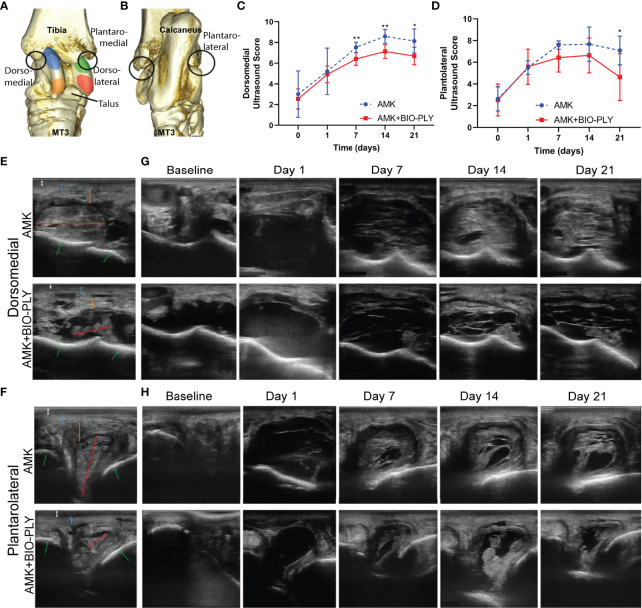
Treatment with AMK+BIO-PLY decreased ultrasound scores and altered appearance at day 7, 14 and 21. Transverse and longitudinal grayscale images of the **(A)** dorsomedial and **(B)** plantolateral recesses of the tarsocrural joint were acquired with the limb weight bearing. **(C, D)** Total ultrasound scores for each recess where 0 = most normal. **(E, F)** Representative transverse images of a control and treatment horse at day 21 labeled for important structures as follows: white double headed arrow = subcutaneous edema/effusion, blue double headed arrow = joint capsule, orange double headed arrow = synovial thickening, red double headed arrow = intra-articular fibrin, green arrows = subchondral bone. **(G-H)**. Representative transverse images of a control and treatment horse at days 0, 1, 7, 14, and 21. Means and standard deviations of each group (control vs treatment; n=6), and significant differences *p<0.05 **p<0.01 were determined by paired t-tests comparing control and treatment at each day. See **Supplementary Figure 3** for individual ultrasound parameter scores and **Supplementary Figure 1** for additional anatomic descriptions of the dorsomedial and plantarolateral recesses of the equine tarsocrural joint.

Using ultrasound to monitor the progression of infectious arthritis (Gaigneux et al., 2017), AMK+BIO-PLY treated horses had an improved ultrasonographic appearance compared to AMK treated horses as evident by changes in joint capsule and synovial thickening as well as the presence of intra-articular fibrin in both the dorsomedial (**Figures 3A, E**). and plantarolateral recesses (**Figures 3B, F**). Over the 21 days, AMK+BIO-PLY treated horses had improved (=lower) ultrasonographic scores compared to AMK horses at days 7 (p<0.007), 14 (p<0.006), and 21 (p<0.02) for the dorsomedial recess (**Figures 3C, G**) and at day 21 (p<0.01) for the plantolateral recess (**Figures 3D, H**). Reduced joint distension (**Supplementary Figure 3A**; p<0.03), reduced synovial thickening (**Supplementary Figure 3B**; p<0.004) and fewer fibrinous loculations within the joint space (**Supplementary Figure 3C**; p<0.03) were evident. No differences in joint effusion (**Supplementary Figure 3D**), intra-articular hyperechoic foci (**Supplementary Figure 3E**) or vascularity (**Supplementary Figure 3F**) were found. Overall, these ultrasonographic changes were consistent with a resolving infection.

### AMK+BIO-PLY Treatment Reduced Joint Neutrophil and Systemic D-Dimer Concentrations

**Figure 4:**
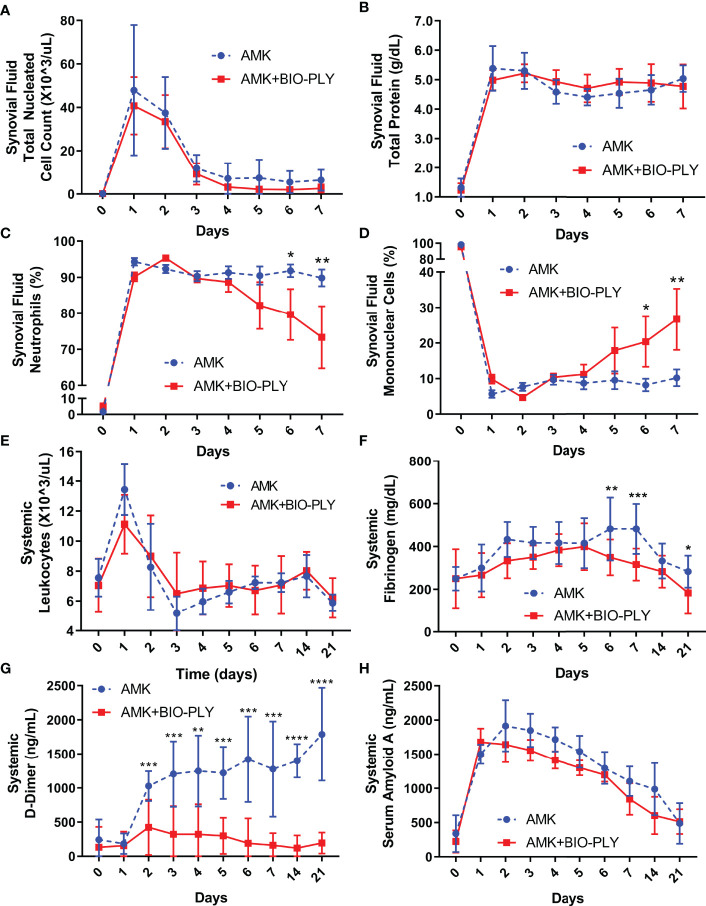
AMK+BIO-PLY treatment altered cellular populations in the synovial fluid and systemic biomarkers. **(A–D)** Synovial fluid total nucleated cell count, total protein, neutrophil percentage, and mononuclear cell percentage. **(E–H)** Systemic leukocyte count, fibrinogen concentration, D-dimer concentration, and Serum Amyloid A concentration. Means and standard deviations of each group (control vs treatment; n=6), and significant differences *p<0.05 **p<0.01 ***p<0.001 ****p<0.0001 were determined by the paired t-tests comparing control and treatment at each day.

No differences in SF total nucleated cell count (**Figure 4A**) or SF total protein content (**Figure 4B**) was found between the groups. The SF percentage of neutrophils (**Figure 4C**) decreased in AMK+BIO-PLY treated horses concomitant with increased percentages of mononuclear cells (**Figure 4D**); these values were significantly different from the AMK group at day 6 and 7 (p<0.02). No differences in systemic leukocyte counts were observed between the two groups throughout the study (**Figure 4E**). Fibrinogen (**Figure 4F**; a protein for blood coagulation) and circulating D-dimer (**Figure 4G**; product of fibrin breakdown) associated with coagulopathy and infection (Shahi et al., 2017; Klim et al., 2018) were decreased with AMK+BIO-PLY treatment compared to the AMK control horses. Serum amyloid A (**Figure 4H**; an acute phase protein) associated with infection and inflammation (Jacobsen et al., 2006) was not different between treatment groups. No alterations in other systemic parameters (i.e., circulating inflammatory cell counts) were observed between treatment groups (**Supplementary Figure 4**). While many markers of inflammation remained similar between the two groups during treatments, synovial neutrophil and systemic D-dimer content were significantly decreased after treatment with AMK+BIO-PLY.

### AMK+BIO-PLY Treatment Altered Synovial Fluid Cytokine Milieu Over Time

**Figure 5:**
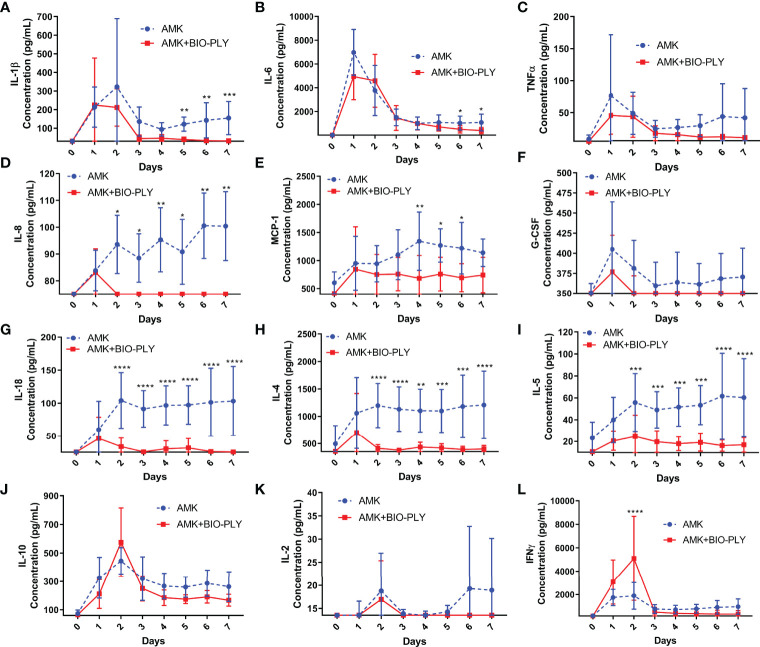
AMK+BIO-PLY treatment altered cytokine parameters in synovial fluid. **(A–L)** Synovial fluid concentration of IL-1β, IL-6, TNFα, IL-8, MCP-1, G-CSF, IL-18, IL-4, IL-5, IL-10, IL-2, IFNγ. For each cytokine, the y-axis starts with the lower limit of detection for that cytokine as defined by the manufacturer of the assay. Means and standard deviations of each group (control vs treatment; n=6), and significant differences *p<0.05 **p<0.01 ***p<0.001 ****p<0.0001 were determined by the paired t-tests comparing control and treatment at each day.

Infected joints respond to microbial challenge by inducing the production of cytokines and chemokines that activate resident macrophages and serve as neutrophil chemoattractants (Davis, 2005). Cytokine levels increased with the onset of infection (**Figure 5**) and the overall increase was moderated by AMK+BIO-PLY treatment, where day 7 levels associated with AMK+BIO-PLY treatment approached the initial day 0 levels. This was not achieved with AMK alone. Specifically, IL-1β (**Figure 5A**) and IL-6 (**Figure 5B**), IL-8 (**Figure 5D**), IL-18 (**Figure 5G**), MCP-1 (**Figure 5E**), IL-4 (**Figure 5H**), and IL-5 (**Figure 5I**) initially increased in all joints but were significantly lower in AMK+BIO-PLY-treated joints compared to AMK treated joints by day 7 (p<0.05). IFNγ (**Figure 5L**) had an initial increase at day 2 in AMK+BIO-PLY horses (p<0.003) but decreased by day 3 to the same level as AMK treated horses. AMK+BIO-PLY treatment did not alter SF concentrations of TNF-α (**Figure 5C**), G-CSF (**Figure 5F**), IL-10 (**Figure 5J**), IL-2 (**Figure 5K**), FGF2 (**Supplementary 5A**), eotaxin (**Supplementary Figure 5B**), GM-CSF (**Supplementary Figure 5C**), IL-1α (**Supplementary Figure 5D**), fractalkine (**Supplementary Figure 5E**), IL-13 (**Supplementary Figure 5F**), IL-17A (**Supplementary Figure 5G**), IL-12p70 (**Supplementary Figure 5H**), IP-10 (**Supplementary Figure 5I**), or GRO (**Supplementary Figure 5J**). Taken together, these cytokine profiles suggest an immune-modulating response for AMK+BIO-PLY in the resolution of infection.

### AMK+BIO-PLY Treatment Decreased Synovial Inflammation, Subintimal Vascularity and Fibrosis

**Figure 6:**
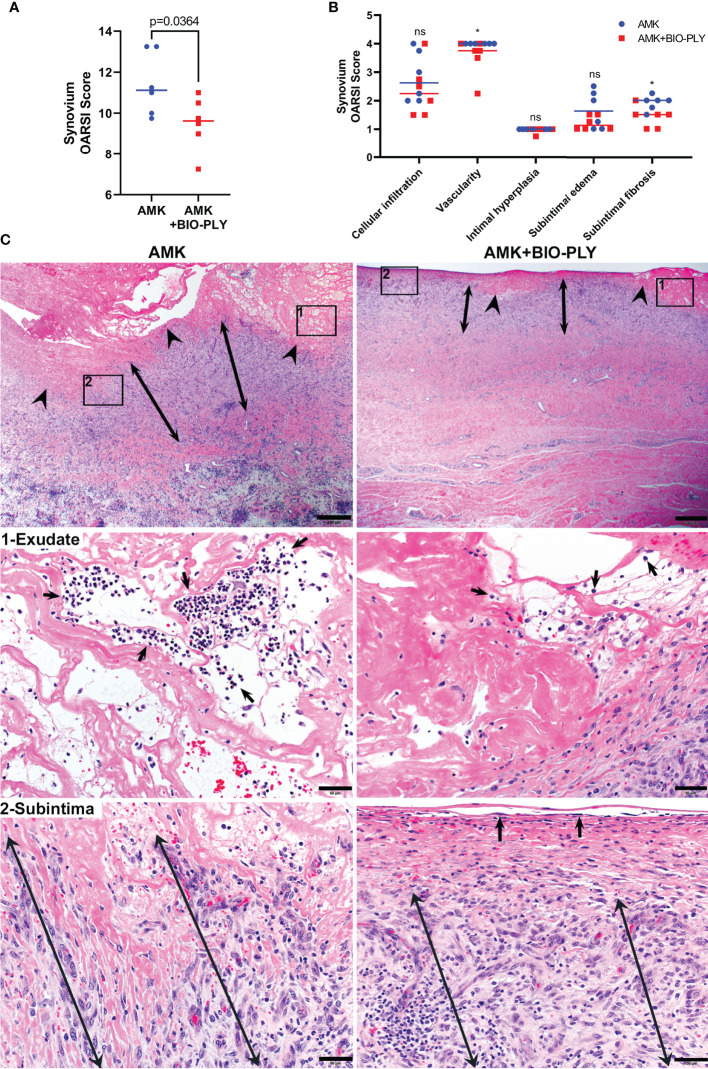
AMK+BIO-PLY treated horses had decreased synovial inflammation and hypertrophy. **(A)** Total OARSI synovial scores. **(B)** Individual components of the synovial OARSI histologic scoring system showed reduction of synovial vascularity and subintimal fibrosis in AMK+BIO-PLY treated compared to AMK control horses. Individual scores with the mean (bar) is shown for each component. ns, no significant difference between AMK and AMK+BIO-PLY; *p < 0.04. **(C)** H&E-stained photomicrographs of synovial sections. Upper panels (scale bars = 500µm) show ulcerated synovial intima (arrowheads) with thicker fibrin exudate (frame-1) that in mid-panels (scale bars = 50µm) shows neutrophilic infiltrates (arrows) corresponding to pockets of neutrophils in AMK treated joints versus rare, individual neutrophils in AMK+BIO-PLY treated joints. Subintimal stroma (double-headed arrows) highlighted in frame-2 and lower panels (scale bars = 50µm) shows in AMK treated joints fibrin interspersed with comparably thicker bands of loose fibrovascular tissue subtending confluent ulcers compared to AMK+BIO-PLY treated joints that show a well-demarcated, compacted band of fibrovascular tissue subtending a surface layer of flattened, regenerating intimal cells (arrows).

The cardinal histologic features of joint infections are synovial inflammation with surface erosions and fibrinosuppurative exudates, subintimal infiltration by neutrophils and mononuclear cells, subintimal neovascularization, and fibrosis (Della Valle et al., 1999). Following euthanasia of horses at day 23-24, a series of postmortem analyses were performed on target tissues from four sites within the tarsocrural joint (**Supplementary Figure 1**). Joints treated with AMK+BIO-PLY had lower (i.e., improved) total scores for synovial lesions based upon the scoring system for horses recommended by the Osteoarthritis Research Society International (OARSI) (McIlwraith et al., 2010) (p<0.04) (**Figure 6A**). When individual components of the grading scheme were assessed, AMK+BIO-PLY treated joints had significantly lower (i.e., less severe) scores for both vascularity and subintimal fibrosis (p<0.04) (**Figure 6B**). Cellular infiltration and subintimal edema trended lower for AMK+BIO-PLY treated joints but were not statistically significant. Because the OARSI scoring system is defined for horses with non-septic osteoarthritis, tissue sections were evaluated for characteristics of septic joints exemplified by fibrin and inflammatory cells within adherent surface exudates as well as the subintima. AMK treated synovium had more extensive intimal ulceration with thick, adherent, poorly organized mats of fibrin with numerous, predominantly neutrophilic, inflammatory cell pockets. In contrast, AMK+BIO-PLY treated joints had less extensive intimal ulcers with scant, better defined, organizing layers of fibrin that contained few, predominantly mononuclear, inflammatory cells (**Figure 6C**, upper and middle panels). AMK treated subintima showed expansion by poorly organized, thick bands of immature fibrovascular tissue with relative increases in inflammatory cells infiltrating deeper layers of collagenous tissue. Within AMK+BIO-PLY treated joints, the subintimal fibrovascular tissue was more organized and compacted beneath a thin regenerating intimal membrane composed of flattened spindle-shaped intimal cells (**Figure 6C** lower panels), corresponding to less severe synovial hypertrophy. Thus, by day 23-24, AMK+BIO-PLY treated joints, based on the improved OARSI scores and histology, showed comparatively less severe fibrinosuppurative inflammation and synovial hypertrophy associated with infectious arthritis.

### AMK+BIO-PLY Treatment Reduced Inflammatory Cell Infiltrates in The Synovium

**Figure 7:**
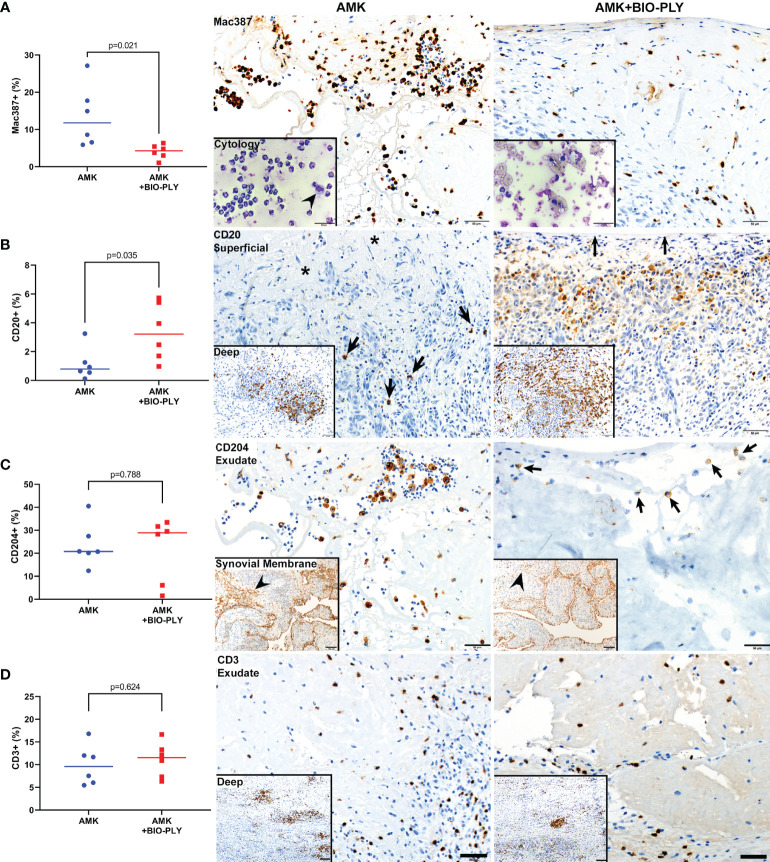
AMK+BIO-PLY treatment altered synovial inflammatory cell infiltrates. Graphs with corresponding photomicrographs of immunohistochemically stained sections of different inflammatory cell populations within synovium from AMK versus AMK+BIO-PLY treated joints. **(A)** Mac387 (myeloid antibody that stains neutrophils and macrophage/monocytes) showed reduced percentage of Mac387+ cells within superficial intimal layers and associated exudates of AMK+BIO-PLY joints (10%) compared to AMK treated joints (90-95%), which corresponded to cytologic smears prepared from synovial fluid sampled at day 7 (insets); where neutrophils surround a single macrophage (arrowhead) in AMK treated synovium compared to 100% mononuclear cells within AMK+BIO-PLY treated synovium; scale bars = 50µm. **(B)** CD20 (B lymphocyte antibody) showed fewer numbers of CD20+ cells (arrows) associated with surface fibrin exudate (asterisks) within superficial subintimal layers of AMK treated joints compared to AMK+BIO-PLY treated joints that have numerous CD20+ cells concentrating within the stroma underlying the intimal surface (arrows); CD20+ cells concentrated within follicular aggregates of the deep subintimal layers (insets, scale bars = 100µm) showed a similar distribution between the two treatment groups. **(C)** CD204+ macrophages showed similar numbers and distribution within exudate and synovial membranes (insets) of both treatment groups; scale bars = 50 µm. **(D)** CD3+ T lymphocytes antibody also showed no differences within synovial exudates (scale bars = 50 µm) or follicular aggregates within deep subintimal stroma (insets; scale bars = 100 µm).

Histochemical and IHC stains were used to analyze synovial inflammation and inflammatory cell populations within superficial intimal and deep subintimal regions of synovial membranes (**Figure 7**) from four representative locations of the tarsocrural joint (**Supplementary Figure 1**). AMK treated synovium had higher numbers and percentages of Mac387+ myeloid leukocytes (*i.e*., neutrophils and monocytes/macrophages) than AMK+BIO-PLY treated synovium (**Figure 7A**). Myeloid leukocytes were concentrated within the superficial intima and adherent fibrinous exudates (p<0.03) but infiltrated all synovial layers. In AMK treated synovium, 90-95% of Mac387+ cells were neutrophils *(i.e*., ~12μm diameter cells with polysegmented nuclei), whereas in AMK+BIO-PLY treated synovium, nearly all Mac387+ cells were mononuclear (*i.e*., monocyte/macrophage). The Mac387 IHC results correlated well with differential cell counts and cytologic slide preparations performed on synovial fluid at day 7 (**Figure 7A**, insets). Increased numbers and percentage of B lymphocytes (CD20+) were identified within superficial synovial layers of AMK+BIO-PLY treated synovium compared to AMK treated controls (p<0.04), although there was variability among individual horses most pronounced in AMK+BIO-PLY treatment group (**Figure 7B**). Numbers and percentages of CD204+ cells (*i.e*., dendritic cells and macrophages) trended higher in AMK+BIO-PLY treated synovial samples, although numbers of CD204+ cells identified within exudate, synovial intimal membranes and subintimal stroma varied among horses representing both treatment groups (**Figure 7C**). No differences in numbers, percentage, or distribution of CD3+ T lymphocytes were identified between the AMK and AMK+BIO-PLY treated groups (**Figure 7D**).

### AMK+BIO-PLY Treatment Exhibited Chondroprotective Effects

**Figure 8:**
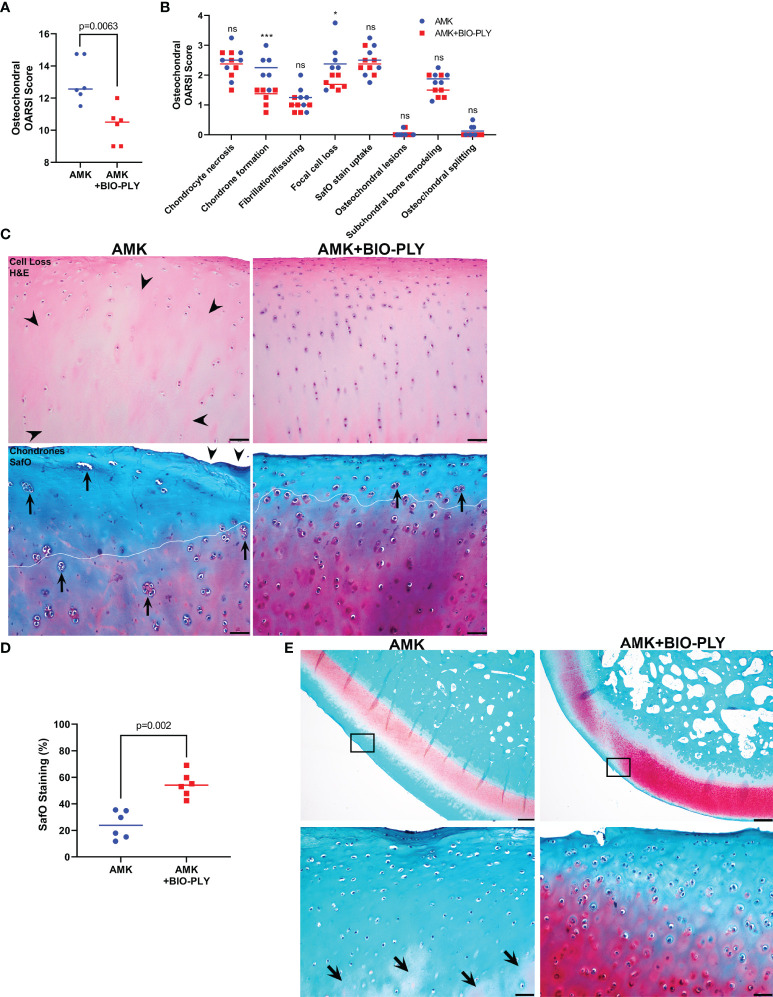
AMK+BIO-PLY treatment mitigated chondrocyte cells loss, chondrone formation and proteoglycan loss within articular cartilage of osteochondral tissues. **(A)** Overall total OARSI scores and **(B)** Individual outcome parameters of the microscopic OARSI scoring system for osteochondral tissue. Individual scores with the mean (bar) is shown for each parameter. ns, no significant difference between AMK and AMK+BIO-PLY; *p<0.02 ***p<0.005. **(C)** H&E stained cartilage showed AMK treated joints had more extensive cartilage cell loss (arrowheads) compared to AMK+BIO-PLY treated joints that retain even distribution of viable chondrocytes. SafO stained cartilage shows more numerous and larger chondrones (arrows) concentrated within increased loss of proteoglycan staining (white line demarcation) in AMK treated joints compared to AMK+BIO-PLY treated horses; scale bars = 50µm. **(D)** Morphometrical analyses of percentage of SafO staining per total area of articular cartilage showed decreased percentage area of SafO staining within non-mineralized cartilage layers in AMK treated joints compared to AMK+BIO-PLY treated joints. **(E)** Representative SafO-stained osteochondral sections with high magnification insets of framed regions from the plantarolateral aspect of the talus highlight loss of proteoglycan staining within AMK treated joints where patchy pale pink staining is reduced to deeper layers of non-mineralized articular cartilage (arrows) compared to AMK+BIO-PLY treated joints; scale bars = 500 µm (upper panels) and 50 µm (lower panels).

Osteochondral explants representing four different locations within the joint were evaluated using the OARSI grading scheme for equine cartilage and subchondral bone (McIlwraith et al., 2010). AMK+BIO-PLY treated osteochondral explants had improved total OARSI scores compared to AMK treated explants (**Figure 8A**; p<0.007). Specifically, when individual components of the grading scheme were compared between groups, scores for focal cell loss (p<0.02) and numbers of chondrocyte clones (i.e., chondrones) (p<0.005) were decreased in AMK+BIO-PLY treated horses as compared to AMK alone (**Figures 8B, C**). Morphometric analyses applied to evaluate the percentage of SafO staining per total area of non-mineralized articular cartilage showed a decreased percentage area of SafO staining (p<0.03) in AMK treated joints compared to AMK+BIO-PLY treated horses (**Figures 8D, E**), indicating that BIO-PLY treatment mitigated inflammation-associated loss of proteoglycan content within the cartilage extracellular matrix. Together these findings suggest that AMK+BIO-PLY mitigates the severity of catabolic tissue response.

## Discussion

This manuscript presents an equine model of *S. aureus* infectious arthritis demonstrating *in vivo* efficacy of BIO-PLY treatment in combination with the aminoglycoside AMK compared to AMK treatment alone. In support of our hypothesis, we demonstrated that AMK+BIO-PLY treatment significantly lowered bacterial concentrations within SF during the 7 days of treatment; at end-term, bacteria were no longer detected in the synovium of the majority of AMK+BIO-PLY treated joints. Overall, the physical appearance of the tarsocrural joints in the treated horses showed improved clinical parameters, including decreased swelling and distal limb edema. A subset of inflammatory markers improved over the 7 days of AMK+BIO-PLY treatment, and histological features observed at end-term confirmed decreased synovial inflammation, including reduced neutrophilic infiltrates within adherent fibrin exudates as well as SF, and reduced early articular cartilage degeneration characterized by fewer chondrones, reduced focal cell loss, and improved cartilage proteoglycan content when compared to AMK treatment alone. It is noteworthy that the AMK+BIO-PLY treatment was able to achieve this outcome in the absence of joint lavage. The findings in this *in vivo* equine study corroborate our previous *in vitro* findings (Gilbertie et al., 2018; Gilbertie et al., 2020) and suggest that AMK+BIO-PLY mitigates equine septic arthritis.

Our choice of an experimental equine model was driven by our extensive experience from clinical equine practice treating horses with infected synovial structures and osteoarthritis (Gilbertie et al., 2018). The experimental equine model is also attractive given that considerable objective information has been generated from this model to understand mechanisms and activity of disease modifying osteoarthritic drugs (McIlwraith et al., 2012). Thus, a therapeutic intervention such as the one discussed in this report would have a direct and immediate benefit to horses in clinical equine practice as well as having the potential to directly translate to human medicine, especially considering most investigations studying the translational merits of anti-infective therapies are limited to small animal models (Corrado et al., 2016).

The poor outcomes associated with joint infection, especially periprosthetic joint infection (PJI) secondary to joint replacement, have recently been suggested to depend on the presence of fibrin-rich floating and adherent antibiotic-tolerant bacterial aggregates (Dastgheyb et al., 2015; Gilbertie et al., 2019; Bidossi et al., 2020). *In vitro*, PRP exhibits antimicrobial activity against planktonic and biofilm bacteria (Drago et al., 2014; López et al., 2014; Zhang et al., 2019; Gilbertie et al., 2020; Attili et al., 2021). *In vivo*, PRP alone or in combination with conventional antimicrobials has been effective in animal models of osteomyelitis and infected wounds (Li et al., 2013; Farghali et al., 2019; Wang et al., 2019; Tang et al., 2021; Wei et al., 2021; Pourkarim et al., 2022). In humans, PRP has also been proven to be safe and efficacious in the clinical treatment of chronic wounds by advancing complete wound closure and reducing wound healing time as well as wound area and depth (Qu et al., 2021). These studies, while promising, often suffer from tremendous variability associated with individual PRP sources. We found that we could increase reproducibility by pooling PRP from multiple animals. Using this pooled PRP, we found that a low molecular weight (<10kDa), cationic PRP fraction showed antimicrobial activity against SF aggregates with synergy achieved in the presence of the aminoglycoside AMK (Gilbertie et al., 2020). Based on this fractionation, it is probable that this PRP-fraction (BIO-PLY) is rich in antimicrobial peptides (AMPs) and that these compounds synergize with AMK to attack both adherent and floating biofilms. The antimicrobial efficacy of the combined AMK+BIO-PLY treatment was validated by the absence of microbial counts in the synovium and the time-dependent reductions in microbial counts in SF compared to AMK alone.

Our previous *in vitro* data indicated a critical role for fibrin in the formation of adherent biofilms and free-floating SF aggregates (Gilbertie et al., 2019; Knott et al., 2021). *S. aureus* can hijack the host coagulation system to aggregate and form biofilms (Crosby et al., 2016), and intra-articular fibrin has been directly associated with increased synovial inflammation and cartilage damage (Flick et al., 2007; Raghu and Flick, 2011). This *in vivo* study showed a marked decrease in ultrasonographically-detected intra-articular fibrinous loculations in the AMK+BIO-PLY treatment group, a finding that correlated with synovial histology. Interestingly, there may be an association between joint inflammation and fibrin(ogen)/coagulation proteins (So et al., 2003; Raghu and Flick, 2011). In models of antigen-induced arthritis (Sanchez-Pernaute et al., 2003), fibrin deposition along the synovial intima, as it is seen in the AMK horses, is associated with synoviocyte activation. In mice lacking fibrinogen or with increased fibrinolysis, fibrin accumulation decreases within their joints resulting in decreased arthritic changes and mitigation of cartilage matrix loss (Flick et al., 2007). In addition, decreased intra-articular fibrin was associated with decreased neutrophil infiltration in the joint of mice lacking fibrinogen (Flick et al., 2007). These studies support the idea that altered fibrin in the joint, whether resulting from bacterial or innate disease processes, is associated with inflammation and joint degradation. Our study showed that AMK+BIO-PLY treated horses had less fibrin deposition that correlated with lower percentages of neutrophils within the SF and tissue. Taken together, our data in the equine model suggest that the reduction in synoviocyte pro-inflammatory cytokine production and increased synoviocyte anti-inflammatory cytokine production (Gilbertie et al., 2018) is reflective of a marked clinical improvement associated with the AMK+BIO-PLY treatment.

In accordance with this data and our ultrasound observations, increased intra-articular fibrinous loculations in the AMK-treated horses correlated with increased serum levels of D-dimers, fibrin degradation products resulting from fibrin dissolution by plasmin (Lee et al., 2018). D-dimer levels of >850 ng/mL have been used to diagnose PJI (Shahi et al., 2017). In our study, AMK+BIO-PLY treatment reduced serum D-dimer levels below 500ng/mL, while AMK horses continued to show elevated D-dimer levels of >1000ng/mL. In addition to serum D-dimer levels, recent studies of PJI biomarkers have proposed serum fibrinogen as a prognostic indicator of infection (Klim et al., 2018), with a range of 361 to 519 mg/dL. In our study, AMK+BIO-PLY treated horses had fibrinogen levels <400 mg/dL while AMK horses had fibrinogen levels >400 mg/dL from day 2 until day 7. Taken together, these findings strongly support the antimicrobial effects of AMK+BIO-PLY in infectious arthritis.

In addition to its antimicrobial effects, AMK+BIO-PLY ameliorated degenerative changes in articular cartilage. Articular cartilage from AMK+BIO-PLY treated horses showed fewer degenerative changes including fewer chondrones, reduced focal cell loss, and reduced loss of cartilage proteoglycan content (i.e., SafO staining) when compared to cartilage from AMK treated joints, consistent with our previous *in vitro* findings (Gilbertie et al., 2018). These parameters may represent early secondary degenerative changes and could have important clinical implications for short-term post-infection recovery and the long-term effects that may prevent chronic inflammation-associated degeneration and preserve joint function.

The levels and ratios of cytokines determine M1/M2 macrophage phenotypes across the continuum of pro-inflammatory and anti-inflammatory states. Horses treated with AMK showed elevated levels of IL-4 and IL-5, consistent with a shift towards the anti-inflammatory M2/Th2 phenotype implicated in biofilm persistence (Hanke and Kielian, 2012). In contrast, horses treated with AMK+BIO-PLY exhibited increased levels of IFNγ followed by decreased IL-4 and IL-5 suggesting a shift towards a pro-inflammatory, M1/Th1 dominant response. Hanke et. al., showed that activated pro-inflammatory macrophages transferred into *S. aureus* biofilms *in vivo* facilitated biofilm clearance, thus further emphasizing the importance of the macrophage activation state (Hanke et al., 2013). IL-18, a cytokine that is constitutively expressed by neutrophils (Robertson et al., 2006), was drastically reduced in AMK+BIO-PLY treated horses. Although IL-18 can induce a Th1 response through the induction of IFNγ, depending on the cytokine environment, it also can induce a Th2 response (Xu et al., 2000). Based on the literature, our data suggest AMK+BIO-PLY treatment appears to facilitate a type 1 response early on and mitigate the inflammatory response at later treatment times. Platelets and platelet-released products are known to interact with the innate and adaptive immune response (Cox et al., 2011); however, future work is required to investigate this relationship.

Neutrophil infiltration and accumulation within the SF and tissue is a hallmark of infectious arthritis (Shirtliff and Mader, 2002). Neutrophils are necessary for the clearance of *S. aureus* during infection; however, their prolonged infiltration can lead to increased joint damage (Boff et al., 2018). Moreover, neutrophils in human knees with non-infectious osteoarthritis are shown as active and synergistic contributors to the progression of inflammation and degeneration in concert with macrophages and lymphocytes (Hsueh et al., 2021). We used the immunohistochemical marker Mac387 (Villiers et al., 2006; Wang et al., 2018) to help identify and quantify neutrophils within these equine synovial tissues. Mac387 (*i.e*., S100A9, MRP-14, calprotectin) is an abundant cytosolic protein in neutrophils and is present in lower amounts within monocytes and subsets of macrophages (Soulas et al., 2011). Although Mac387 is associated with increased synovial activation and joint damage in arthritis models (van Lent et al., 2012), it is also used as a biomarker to distinguish infectious arthritis from other inflammatory forms of arthritis (Baillet et al., 2019). AMK+BIO-PLY treated joints had decreases in Mac387 and relative increases in B lymphocytes (CD20) within the subintimal stroma compared to AMK controls, which provides evidence of AMK+BIO-PLY’s potential immunomodulatory effects. In addition to reducing neutrophils within synovial exudates, BIO-PLY also decreased neutrophil associated chemokines and cytokines within the synovial fluid such as IL-8, G-CSF, and MCP-1 (Tecchio et al., 2014). Although CD204, a marker used to detect M2 subsets of macrophages (Heusinkveld and van der Burg, 2011), showed individual variation in numbers and percentages of cells within joints representing both treatment groups, this marker showed high affinity for cells lining synovial intima as well as mononuclear cells within exudates and subintimal stroma. Further investigations are needed to determine the potential role of these cells as well as different populations of lymphocytes in the contribution or resolution of synovial inflammation and cartilage degeneration.

In summary, our data suggests that the persistent molecular and cellular immune response (i.e., pro-inflammatory cytokine milieu corresponding to fibrinosuppurative inflammation and synovial hypertrophy) in AMK-treated horses is detrimental to articular cartilage. In contrast, the coordinated changes in the immune response of AMK+BIO-PLY-treated horses are consistent with an early clearance of bacterial biofilm that is supported by the decreased synovial bacterial counts and our *in vitro* studies that mitigate persistent fibrinosuppurative inflammation and associated cartilage degeneration. Limitations of this study include lack of data from a BIO-PLY alone treatment group and the assessment of acute (within 24 hours) infectious arthritis due to *S. aureus* only. Nevertheless, we conclude that intra-articular treatment with this antibiofilm, platelet-rich-plasma derived cationic fraction represents a powerful approach for augmenting treatment of *S. aureus* infectious arthritis.

## Data Availability Statement

The raw data supporting the conclusions of this article will be made available by the authors, without undue reservation.

## Ethics Statement

The animal study was reviewed and approved by Institutional Animal Care and Use Committee of North Carolina State University.

## Author Contributions

All authors contributed to study conception and design. JG, TS, JE, GS, BD, AS, GR, DS, AF, and LS contributed to acquisition of the data. DS performed the statical analysis. All authors contributed to interpretation of the data. JG drafted the manuscript with all authors contributing edits. All authors contributed to the article and approved the submitted version.

## Funding

This work was supported by the Raymond Firestone Trust (JG and TS), University of Pennsylvania Internal Grant (JG and TS), American Quarter Horse Foundation (JG, TS, and LS), Grayson-Jockey Club Research Foundation, Inc. (JG, TS, and LS), Morris Animal Foundation (JG and LS) and the Fund for Orthopedic Research in honor of Gus and Equine athletes (F.O.R.G.E; LS). Work reported in this publication was partially supported by the National Institutes of Health (NIH) under award numbers R01 AR069119 and R01 AR076941 (NH, TS). The content is solely the responsibility of the authors and does not necessarily represent the official views of the National Institutes of Health.

## Conflict of Interest

The contents of this manuscript are the subject of a patent filed by NC State University and the University of Pennsylvania by the authors (JG, TS, and LS).

The authors declare that the research was conducted in the absence of any commercial or financial relationships that could be construed as a potential conflict of interest.

## Publisher’s Note

All claims expressed in this article are solely those of the authors and do not necessarily represent those of their affiliated organizations, or those of the publisher, the editors and the reviewers. Any product that may be evaluated in this article, or claim that may be made by its manufacturer, is not guaranteed or endorsed by the publisher.

## Supplementary Material

Supplementary Figure 1 Anatomic descriptions of the equine tarsocrural joint. Anatomic locations labeled on a three-dimensional computed tomography (CT) rendering of a healthy equine tarsal joint from **(A)** dorsal and **(D)** plantar aspects. Individual bones and the recesses are labeled or circled respectively. The colored areas are the locations that osteochondral sections were collected. Blue=plantaromedial, green=plantarolateral, red=dorsolateral, orange=dorsomedial. Synovium was collected adjacent to the indicated osteochondral locations. MT, metatarsal, T=tarsal; CT, central tarsal. Anatomic locations of the **(B, C)** dorsomedial and **(E, F)** plantarolateral recesses of the equine tarsocrural joint as shown on Magnetic Resonance Imaging (MRI). **(A)** Coronal plane VIBE MRI image highlighting the dorsomedial recess (red arrow). **(B)** Transverse plane fat saturated PD MRI image highlighting the dorsomedial recess with high signal intensity (white) synovial fluid (red arrow) at the level of the dotted red line shown in **(A)**. **(C)** Sagittal plane fat saturated MRI image highlighting the plantarolateral recess (red arrow). **(D)** Transverse plane fat saturated PD MRI image highlighting the plantarolateral recess with high signal intensity (white) synovial fluid (red arrow) at the level of the dotted red line shown in **(C)**.

Supplementary Figure 2 Enzymatic dispersal of infected synovial fluid. Synovial fluid was collected and pooled from 4 horses and subsequently infected ex vivo with S. aureus (ATCC 25923) at 1x105 CFU/mL. **(A)** Infected synovial fluid was left untreated, sonicated for 1 hr in an ultrasonic bath, or treated with proteinase K (200µg/mL) for 15 minutes. **(B)** Synovial fluid was then serial diluted, and spot plated for **(C)** bacterial enumeration as CFU/mL.

Supplementary Figure 3 Individual ultrasound parameter scores. Ultrasound images were assessed using established criteria for infectious arthritis with each category scored on a scale of 0-3 (0 = most normal, 3 = most abnormal): degree of distension **(A)** degree of synovial thickening **(B)** presence of fibrinous loculations **(C)** character of synovial effusion **(D)** presence of hyperechoic spots **(E)** and vascularity by Doppler **(F)**. Means and standard deviations of each group (control vs treatment; n=6), and significant differences *p<0.05 were determined by the paired t-tests comparing control and treatment at each day. Data shown here are the individual parameters of the total ultrasound scores shown in **Figure 5**.

Supplementary Figure 4 Additional complete blood count parameters. **(A-C)** Systemic neutrophil, lymphocyte, and monocyte counts. Means and standard deviations of each group (control vs treatment; n=6), were determined by the paired t-tests comparing control and treatment at each day; no significant differences were found.

Supplementary Figure 5 Additional cytokine concentrations in synovial fluid. **(A-J)** Synovial fluid concentration of FGF2, eotaxin, GM-CSF, IL-1α, fractalkine, IL-13, IL-17A, IL-12p70, IP-10, and GRO. For each cytokine, the y-axis starts with the lower limit of detection for that cytokine as defined by the manufacturer of the assay. Means and standard deviations of each group (control vs treatment; n=6) red t-tests comparing control and treatment at each day; no significant differences were found.
