# Supplementary figures and images for: A Platelet-Rich Plasma-Derived Biologic Clears Staphylococcus aureus Biofilms While Mitigating Cartilage Degeneration and Joint Inflammation in a Clinically Relevant Large Animal Infectious Arthritis Model
Source: Front Cell Infect Microbiol. 2022 May 30;12:895022. doi: 10.3389/fcimb.2022.895022 (PMC9195519; doi:10.3389/fcimb.2022.895022)

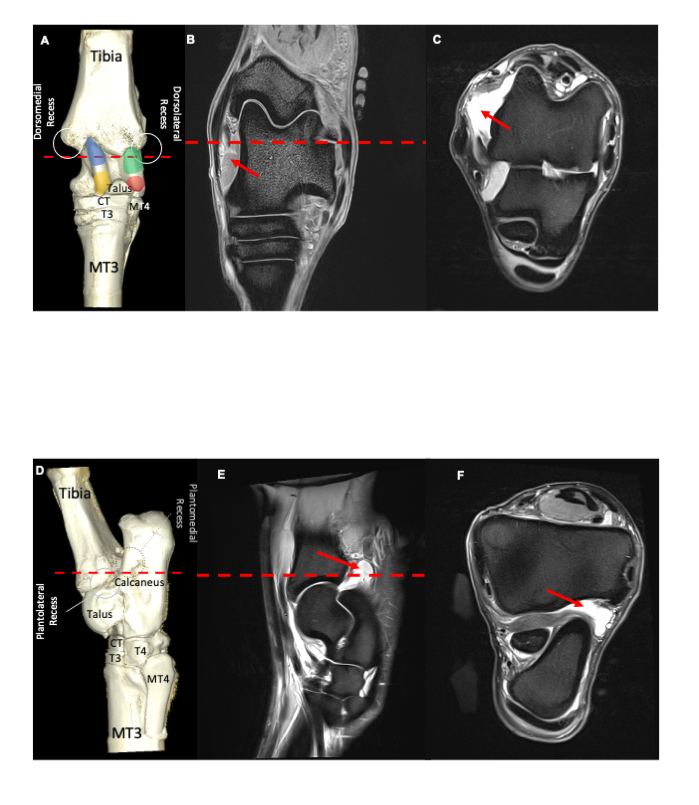

Supplement: Supplementary Figure 1 — Anatomic descriptions of the equine tarsocrural joint. Anatomic locations labeled on a three-dimensional computed tomography (CT) rendering of a healthy equine tarsal joint from (A) dorsal and (D) plantar aspects. Individual bones and the recesses are labeled or circled respectively. The colored areas are the locations that osteochondral sections were collected. Blue=plantaromedial, green=plantarolateral, red=dorsolateral, orange=dorsomedial. Synovium was collected adjacent to the indicated osteochondral locations. MT, metatarsal, T=tarsal; CT, central tarsal. Anatomic locations of the (B, C) dorsomedial and (E, F) plantarolateral recesses of the equine tarsocrural joint as shown on Magnetic Resonance Imaging (MRI). (A) Coronal plane VIBE MRI image highlighting the dorsomedial recess (red arrow). (B) Transverse plane fat saturated PD MRI image highlighting the dorsomedial recess with high signal intensity (white) synovial fluid (red arrow) at the level of the dotted red line shown in (A). (C) Sagittal plane fat saturated MRI image highlighting the plantarolateral recess (red arrow). (D) Transverse plane fat saturated PD MRI image highlighting the plantarolateral recess with high signal intensity (white) synovial fluid (red arrow) at the level of the dotted red line shown in (C). [file Image_1.tiff]

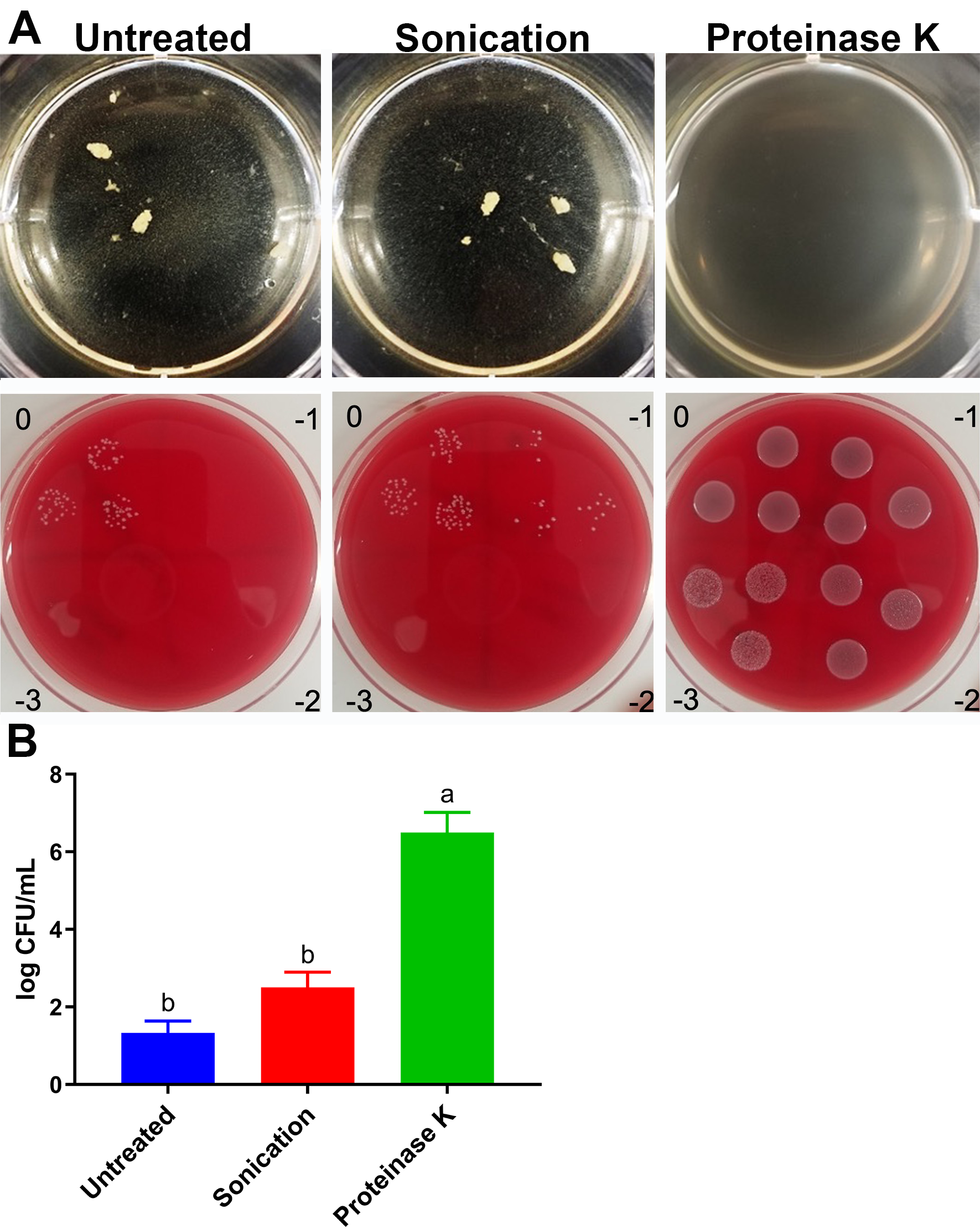

Supplement: Supplementary Figure 2 — Enzymatic dispersal of infected synovial fluid. Synovial fluid was collected and pooled from 4 horses and subsequently infected ex vivo with S. aureus (ATCC 25923) at 1x10^5^ CFU/mL. (A) Infected synovial fluid was left untreated, sonicated for 1 hr in an ultrasonic bath, or treated with proteinase K (200µg/mL) for 15 minutes. (B) Synovial fluid was then serial diluted, and spot plated for (C) bacterial enumeration as CFU/mL. [file Image_2.tif]

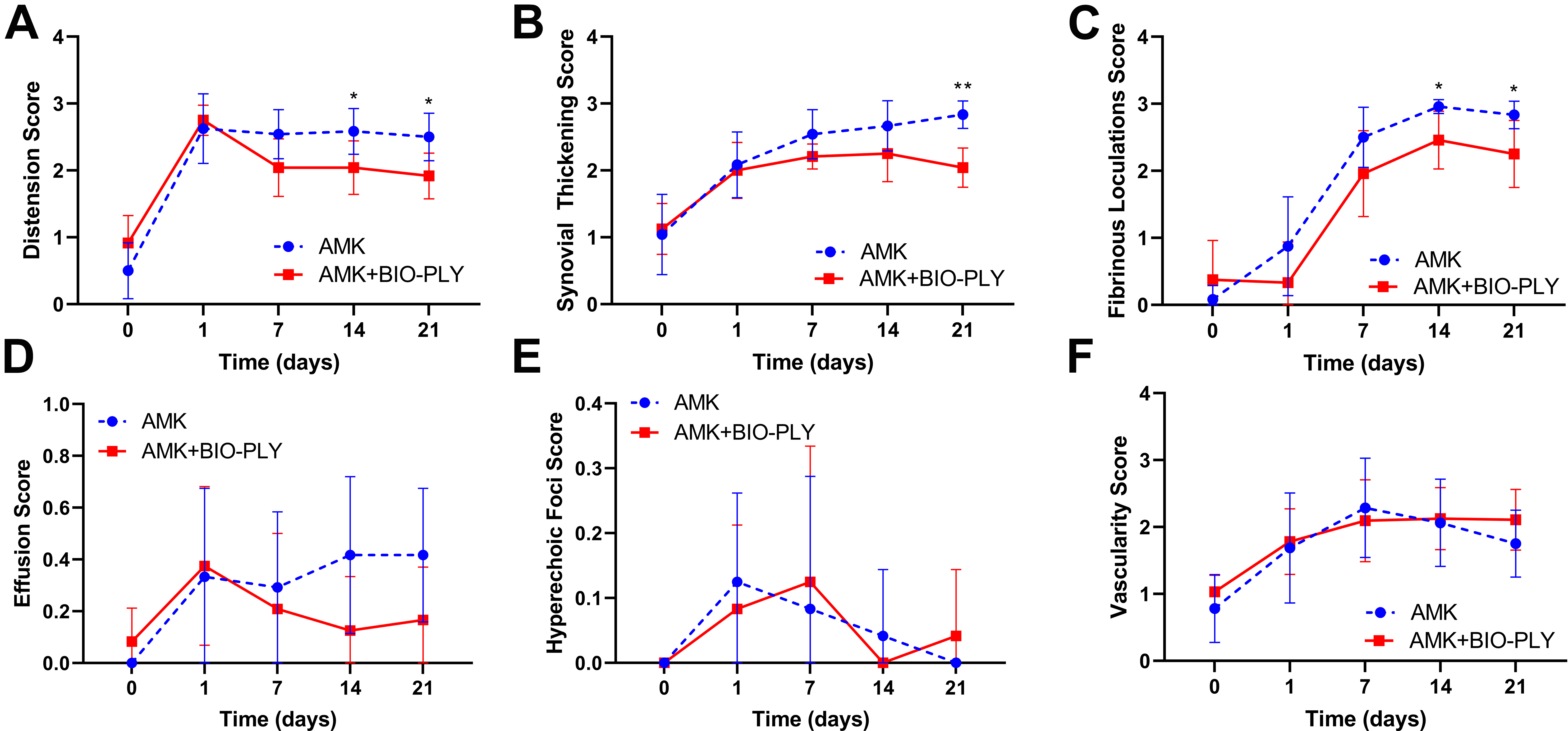

Supplement: Supplementary Figure 3 — Individual ultrasound parameter scores. Ultrasound images were assessed using established criteria for infectious arthritis with each category scored on a scale of 0-3 (0 = most normal, 3 = most abnormal): degree of distension (A) degree of synovial thickening (B) presence of fibrinous loculations (C) character of synovial effusion (D) presence of hyperechoic spots (E) and vascularity by Doppler (F). Means and standard deviations of each group (control vs treatment; n=6), and significant differences *p<0.05 were determined by the paired t-tests comparing control and treatment at each day. Data shown here are the individual parameters of the total ultrasound scores shown in Figure 5 . [file Image_3.tif]

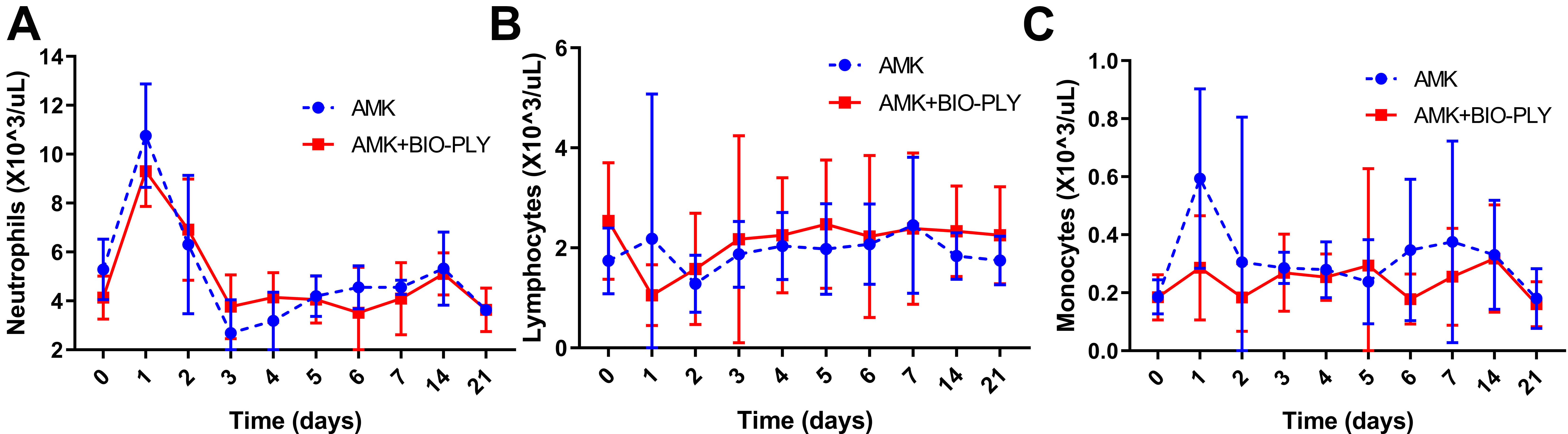

Supplement: Supplementary Figure 4 — Additional complete blood count parameters. (A-C) Systemic neutrophil, lymphocyte, and monocyte counts. Means and standard deviations of each group (control vs treatment; n=6), were determined by the paired t-tests comparing control and treatment at each day; no significant differences were found. [file Image_4.tif]

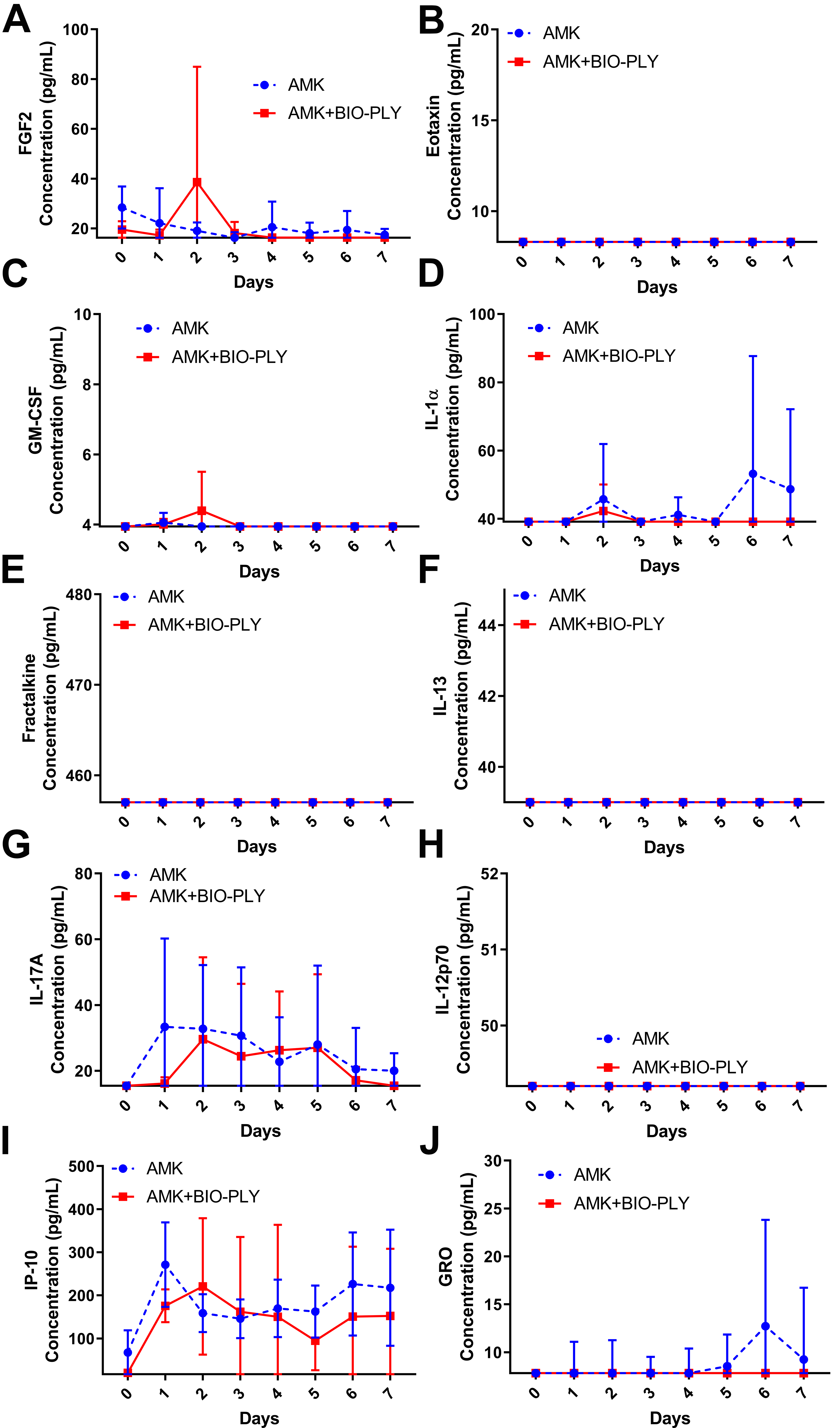

Supplement: Supplementary Figure 5 — Additional cytokine concentrations in synovial fluid. (A-J) Synovial fluid concentration of FGF2, eotaxin, GM-CSF, IL-1α, fractalkine, IL-13, IL-17A, IL-12p70, IP-10, and GRO. For each cytokine, the y-axis starts with the lower limit of detection for that cytokine as defined by the manufacturer of the assay. Means and standard deviations of each group (control vs treatment; n=6) red t-tests comparing control and treatment at each day; no significant differences were found. [file Image_5.tif]
